# Trastuzumab deruxtecan rechallenge in HER2-positive metastatic breast cancer: EN-SEMBLE

**DOI:** 10.1016/j.breast.2026.104883

**Published:** 2026-07-22

**Authors:** Kazuki Nozawa, Hiroji Iwata, Toru Mukohara, Tetsuhiko Taira, Akiyo Yoshimura, Shigenori E. Nagai, Jun Hashimoto, Kazuo Matsuura, Toshiro Mizuno, Yoshiaki Shinden, Mitsugu Yamamoto, Toshimi Takano, Makoto Wakahara, Hirofumi Terakawa, Takashi Yamanaka, Yasuyuki Kojima, Takahiro Nakayama, Yuji Hirakawa, Kazuhito Shiosakai, Junji Tsurutani

**Affiliations:** a Department of Advanced Clinical Research and Development, Nagoya City University Graduate School of Medical Sciences, 1 Kawasumi, Mizuho-cho, Mizuho-ku, Nagoya, Aichi, 467-8601, Japan; b Department of Breast Surgery, Nagoya City University Graduate School of Medical Sciences, 1 Kawasumi, Mizuho-cho, Mizuho-ku, Nagoya, Aichi, 467-8601, Japan; c Department of Medical Oncology, National Cancer Center Hospital East, 6-5-1 Kashiwanoha, Kashiwa, Chiba, 277-8577, Japan; d Department of Oncology, Sagara Hospital, 3-31 Matsubara-cho, Kagoshima, Kagoshima, 892-0833, Japan; e Department of Breast Oncology, Aichi Cancer Center, 1-1 Kanokoden, Chikusa-ku, Nagoya, Aichi, 464-8681, Japan; f Division of Breast Oncology, Saitama Cancer Center, 780 Komuro, Inamachi, Kita-Adachi-gun, Saitama, 362-0806, Japan; g Division of Medical Oncology, St. Luke's International Hospital, 9-1 Akashi-cho, Chuo-ku, Tokyo, 104-8560, Japan; h Department of Breast Oncology, Saitama Medical University International Medical Center, 1397-1 Yamane, Hidaka, Saitama, 350-1298, Japan; i Department of Medical Oncology, Mie University Hospital, 2-174 Edobashi, Tsu, Mie, 514-8507, Japan; j Department of Breast and Thyroid Surgery, Kagoshima University Graduate School of Medical and Dental Sciences, 8-35-1 Sakuragaoka, Kagoshima, Kagoshima, 890-8520, Japan; k Department of Breast Oncology, Hokkaido Cancer Center, 2-3-54 Kikusui Shi-jo, Shiroishi-ku, Sapporo, Hokkaido, 003-0804, Japan; l Department of Breast Medical Oncology, Cancer Institute Hospital of Japanese Foundation for Cancer Research, 3-8-31 Ariake, Koto-ku, Tokyo, 135-8550, Japan; m Department of Breast and Endocrine Surgery, Tottori University Hospital, 36-1 Nishi-cho, Yonago, Tottori, 683-8504, Japan; n Department of Breast Surgery, Kanazawa University Hospital, 13-1 Takara-machi, Kanazawa, Ishikawa, 920-8641, Japan; o Department of Breast Surgery and Oncology, Kanagawa Cancer Center, 2-3-2 Nakao, Asahi-ku, Yokohama, Kanagawa, 241-8515, Japan; p Institute for Clinical Genetics and Genomics, Showa Medical University, 1-5-8 Hatanodai, Shinagawa-ku, Tokyo, 142-8555, Japan; q Department of Breast and Endocrine Surgery, Osaka International Cancer Institute, 3-1-69 Otemae, Chuo-ku, Osaka, Osaka, 541-8567, Japan; r Oncology Medical Science Department I, Daiichi Sankyo Co., Ltd., 3-5-1 Nihonbashi-honcho, Chuo-ku, Tokyo, 103-8426, Japan; s Data Intelligence Department, Daiichi Sankyo Co., Ltd., 1-2-58 Hiromachi, Shinagawa-ku, Tokyo, 140-8710, Japan; t Advanced Cancer Translational Research Institute, Showa Medical University, 1-5-8 Hatanodai, Shinagawa-ku, Tokyo, 142-8555, Japan

**Keywords:** Effectiveness, Human epidermal growth factor receptor 2, Interstitial lung disease, Metastatic breast cancer, Real-world, Trastuzumab deruxtecan rechallenge

## Abstract

**Background:**

Trastuzumab deruxtecan (T-DXd) is a key treatment for HER2-positive metastatic breast cancer (mBC); however, data on T-DXd rechallenge remain limited.

**Patients and methods:**

This post-hoc subgroup analysis evaluated T-DXd rechallenge in patients with HER2-positive mBC using data from EN-SEMBLE, a nationwide cohort study in Japan (N = 664).

**Results:**

Twenty-six patients were included. Median real-world progression-free survival, real-world time to treatment failure, and overall survival were 6.5, 4.9, and 14.2 months, respectively. The objective response rate was 27%. Patients who discontinued initial T-DXd without progressive disease had numerically longer real-world progression-free survival. No interstitial lung disease occurred during rechallenge.

**Conclusion:**

T-DXd rechallenge may have clinical activity in selected patients with HER2-positive mBC.

**Clinical trial registration number:**

jRCT1030220506.

## Highlights

• T-DXd rechallenge was evaluated after one non-T-DXd intervening treatment line.
• Median rwPFS/OS during T-DXd rechallenge were 6.5/14.2 months, respectively.
• Objective responses were observed in 27% of patients receiving T-DXd rechallenge.
• Patients without PD on initial T-DXd had numerically longer effectiveness outcomes.
• No ILD occurred during T-DXd rechallenge in the small cohort, including in patients with prior ILD.

## Graphical abstract

**Figure:**
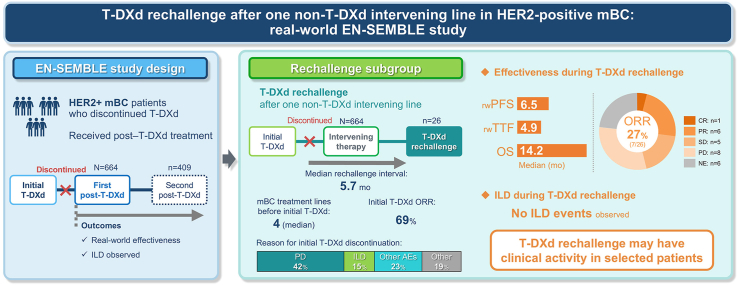

**Abbreviations**
ADC: antibody–drug conjugate
AE: adverse event
BC: breast cancer
BOR: best overall response
CDK4/6i: cyclin-dependent kinase 4/6 inhibitor
CI: confidence interval
CR: complete response
DOT: duration of treatment
ECOG PS: Eastern Cooperative Oncology Group performance status
HER2: human epidermal growth factor receptor 2
IHC: immunohistochemistry
ILD: interstitial lung disease
ISH: in situ hybridization
mBC: metastatic breast cancer
mo: month
NC: not calculated
NE: not evaluable
No: number
ORR: objective response rate
OS: overall survival
PD: progressive disease
PMS: post-marketing surveillance
PR: partial response
Pt: patient
Q: quartile
rwPFS: real-world progression-free survival
rwTTF: real-world time to treatment failure
SD: stable disease
T-DM1: trastuzumab emtansine
T-DXd: trastuzumab deruxtecan
TKI: tyrosine kinase inhibitor
Tx: treatment

## 1 Introduction

The treatment landscape for human epidermal growth factor receptor 2 (HER2)-positive metastatic breast cancer (mBC) continues to evolve, with trastuzumab deruxtecan (T-DXd) moving from later-line settings to standard second-line treatment and earlier therapy lines [[1], [2], [3], [4], [5]]. In parallel, maintenance strategies after first-line induction treatment are gaining attention, further highlighting the importance of optimizing treatment sequencing after T-DXd. While reports have assessed therapies after T-DXd discontinuation [6,7], evidence on T-DXd re-administration (i.e., rechallenge), particularly after an intervening non-T-DXd treatment line, remains limited. Consequently, important clinical questions remain, including how to select patients for rechallenge, whether rechallenge is effective, which populations may benefit, and the tolerability of rechallenge in patients who discontinued T-DXd because of adverse events (AEs).

EN-SEMBLE was a real-world, nationwide cohort study in Japan examining the distribution and outcomes of subsequent treatments after T-DXd discontinuation in 664 patients with HER2-positive mBC [6,8]. The present analysis explored the effectiveness and interstitial lung disease (ILD) incidence associated with T-DXd rechallenge after one intervening line of non-T-DXd treatment within the EN-SEMBLE study.

## 2 Materials and methods

### 2.1 Study design

Full details of the study design and primary results have been published [6]. This post hoc subgroup analysis included patients enrolled in EN-SEMBLE (jRCT1030220506) who underwent T-DXd rechallenge as their second post-T-DXd treatment after one non-T-DXd intervening line. EN-SEMBLE only collected data on the first and second treatment lines after initial T-DXd discontinuation. Thus, outcomes among patients who received non-T-DXd therapy at the same post-T-DXd treatment line were summarized for descriptive purposes only. No direct comparative analyses were planned or performed.

### 2.2 Ethics

EN-SEMBLE was conducted in accordance with the Declaration of Helsinki [9] and the relevant Japanese ordinance and approved by the Showa University Research Ethics Review Board. All participants provided written informed consent or were allowed to opt out.

### 2.3 Outcomes

Treatment effectiveness (real-world progression-free survival [rwPFS], real-world time to treatment failure [rwTTF], and overall survival [OS]) was evaluated using the start date of T-DXd rechallenge as the index date. Disease progression, best overall response (BOR), reason for initial T-DXd discontinuation, and ILD incidence were assessed by treating physicians in routine clinical practice; objective response rate (ORR) was calculated based on BOR [6].

### 2.4 Statistical analysis

The median rwPFS, rwTTF, and OS and their 95% confidence intervals (CIs) were estimated using the Kaplan–Meier and Brookmeyer–Crowley methods, respectively, and stratified by BOR during initial T-DXd treatment and by reason for initial T-DXd treatment discontinuation. Missing data were not imputed. Analyses were performed using SAS v9.4 (SAS Institute, Inc.).

## 3 Results

### 3.1 Patients

**Fig. 1:**
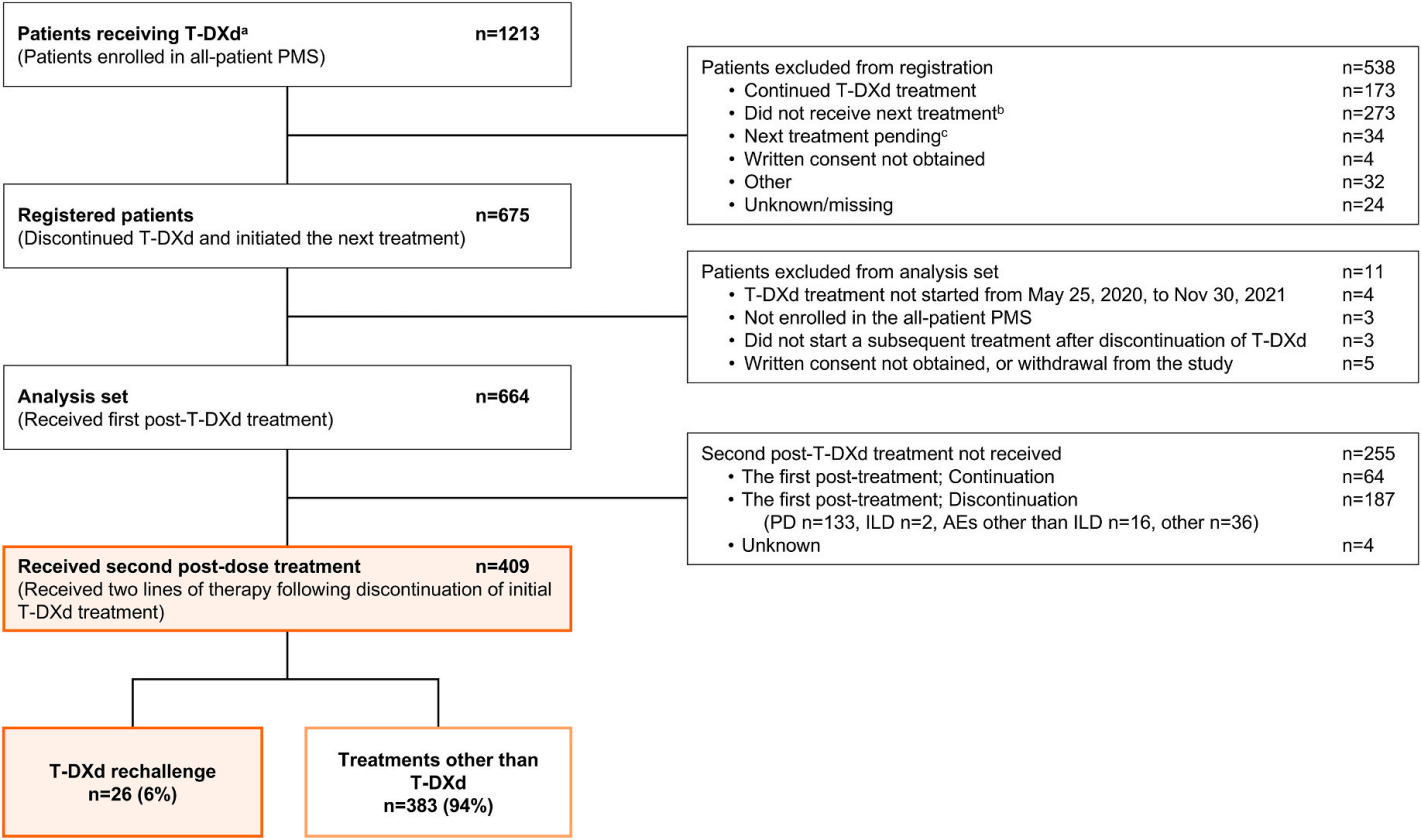
Patient disposition ^a^Among these, 3 patients started administration of T-DXd on or after December 1, 2021. ^b^Death, palliative care, lost to follow-up, transferred to another hospital after discontinuation of T-DXd. cNot started next treatment after discontinuation of T-DXd.

Of 664 patients in the EN-SEMBLE study [6], 409 received both first and second post-T-DXd treatment after initial T-DXd discontinuation (Fig. 1). Of those, 26 (6%) received T-DXd rechallenge after one non-T-DXd intervening line and were included in the analysis (single-agent T-DXd, n = 25; T-DXd plus fulvestrant, n = 1). The remaining 383 received treatment other than T-DXd as their second post-T-DXd treatment. Among rechallenge patients, the median (Q1, Q3) number of treatment lines for mBC before initial T-DXd treatment was 4 (3, 5) and the median interval between discontinuation of initial T-DXd treatment and initiation of T-DXd rechallenge was 5.7 months (Q1, Q3: 3.7, 11.4; Table 1). Treatment with initial T-DXd achieved an ORR of 69% (18/26) and was discontinued due to progressive disease (PD; 42% [11/26]), ILD (15% [4/26]), AEs other than ILD (23% [6/26]), and other causes (19% [5/26]) (Table 1). Background and clinical characteristics of these 26 patients are shown in Supplementary Table S1.

**Table 1:** Patient demographic and clinical characteristics.

Items	Patients with T-DXd rechallenge (N = 26)
**At the start of the first post-T-DXd treatment**	
Age	
Median (Q1, Q3), years	56.5 (50, 67)
<65 years	18 (69)
≥65 years	8 (31)
Sex	
Female	26 (100)
Hormone receptor status	
Positive	18 (69)
Negative	8 (31)
HER2 status	
IHC 3+	17 (65)
IHC 2+/ISH+	7 (27)
Unknown/both missing	2 (8)
ECOG PS	
0	14 (54)
1	7 (27)
≥2	3 (12)
Unknown	2 (8)
Visceral metastasis	
Yes	22 (85)
No	4 (15)
Brain metastasis	
Yes	9 (35)
No	17 (65)
**Prior cancer therapy for mBC before initial T-DXd treatment**	
Number of prior regimens	
Median (Q1, Q3), line	4 (3, 5)
≤2 lines	3 (12)
≥3 lines	22 (85)
Unknown/missing	1 (4)
Prior regimens	
Anti-HER2 therapy	25 (96)
Trastuzumab	23 (89)
Pertuzumab	21 (81)
T-DM1	25 (96)
HER2-TKI	11 (42)
Chemotherapy	24 (92)
Endocrine therapy	13 (50)
**Initial T-DXd treatment**	
mBC status	
*De novo* stage IV	3 (12)
Recurrent BC	21 (81)
Others	1 (4)
Unknown/missing	1 (4)
Duration of initial T-DXd treatment, median (Q1, Q3) month	10.6 (8.5, 14.6)
ORR of initial T-DXd treatment	18 (69)
BOR of initial T-DXd treatment	
CR	4 (15)
PR	14 (54)
SD	5 (19)
PD	1 (4)
NE	2 (8)
History of ILD incidence caused by initial T-DXd treatment	
Yes	4 (15)
No	22 (85)
Reason for discontinuation of initial T-DXd	
PD	11 (42)
ILD	4 (15)
AEs other than ILD	6 (23)
Others	5 (19)
Interval between initial T-DXd discontinuation and rechallenge, median (Q1, Q3), months	5.7 (3.7, 11.4)

Data are shown as n (%) unless otherwise noted.

Abbreviations: AE, adverse event; BC, breast cancer; BOR, best overall response; CR, complete response; ECOG PS, Eastern Cooperative Oncology Group performance status; HER2, human epidermal growth factor receptor 2; IHC, immunohistochemistry; ILD, interstitial lung disease; ISH, in situ hybridization; mBC, metastatic breast cancer; NE, not evaluable; ORR, objective response rate; PD, progressive disease; PR, partial response; Q, quartile; SD, stable disease; T-DM1, trastuzumab emtansine; T-DXd, trastuzumab deruxtecan; TKI, tyrosine kinase inhibitor.

Demographic and clinical characteristics of 383 patients who received treatments other than T-DXd are shown in Supplementary Table S2. Among patients who did not undergo rechallenge, ORR to initial T-DXd was 57%, and initial T-DXd was discontinued due to PD in 72% and ILD in 23%.

### 3.2 Effectiveness outcomes

**Fig. 2:**
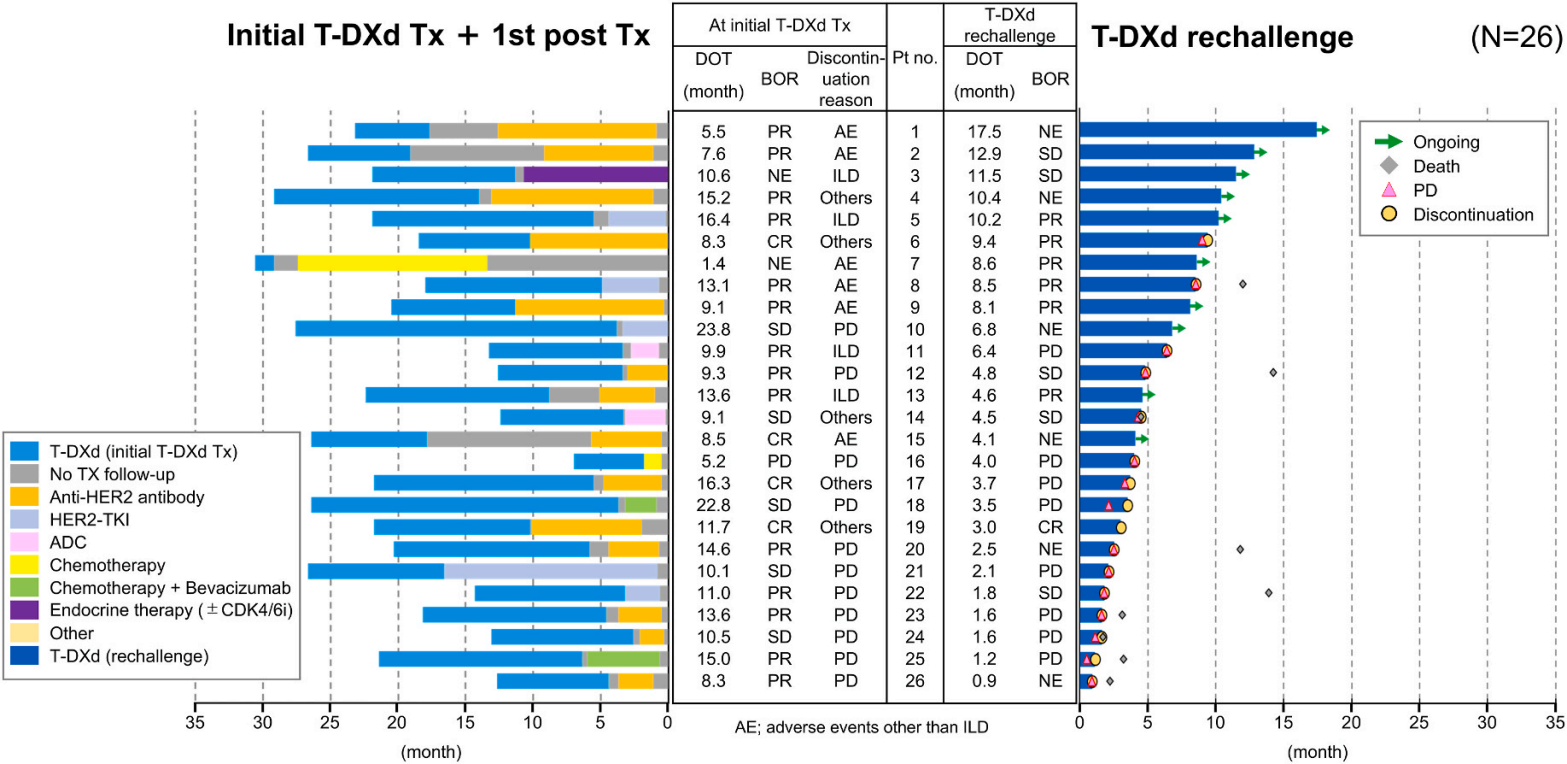
Swimmer plot of treatment and efficacy during the initial T-DXd, first post-T-DXd treatment, and T-DXd rechallenge (N = 26).

Median (Q1, Q3) follow-up from the start of T-DXd rechallenge was 10.3 (4.6, 12.9) months, and 38% (10/26) of patients remained on T-DXd rechallenge treatment at the data cutoff (November 30, 2023). Median rwPFS was 6.5 months (95% CI, 2.5–not calculated [NC]), median rwTTF was 4.9 months (3.0–NC), and median OS was 14.2 months (11.8–NC). The ORR was 27% (7/26) (Table 2). Fig. 2 and Supplementary Table S3 show treatment courses for individual patients, including initial T-DXd duration, intervening therapies and their durations, and T-DXd rechallenge duration. Outcomes for patients receiving treatments other than T-DXd are shown in Supplementary Table S4 for descriptive context only.

**Table 2:** T-DXd rechallenge outcomes.

Items	Patients with T-DXd rechallenge (N = 26)
**Follow-up period, median (Q1, Q3) months**^a^	10.3 (4.6, 12.9)
**Situation at the time of data cutoff, n (%)**	
Continuation	10 (38)
Discontinuation	16 (62)
**Survival outcomes, median (95% CI) months**^a^	
**rwPFS**	
Overall (N = 26)	6.5 (2.5–NC)
By BOR of initial T-DXd	
CR/PR (n = 18)	8.5 (2.5–NC)
SD/PD/NE (n = 8)	4.2(1.1–NC)
By reason for discontinuation of initial T-DXd	
PD (n = 11)	2.1 (0.9–4.0)
ILD, AEs other than ILD, others (n = 15)	Not reached
**rwTTF**	
Overall (N = 26)	4.9 (3.0–NC)
By BOR of initial T-DXd	
CR/PR (n = 18)	6.5 (2.5–NC)
SD/PD/NE (n = 8)	4.3 (1.6–NC)
By reason for discontinuation of initial T-DXd	
PD (n = 11)	2.1 (1.2–4.0)
ILD, AEs other than ILD, others (n = 15)	Not reached
**OS**	
Overall (N = 26)	14.2 (11.8–NC)
By BOR of initial T-DXd	
CR/PR (n = 18)	14.2 (11.8–NC)
SD/PD/NE (n = 8)	Not reached
By reason for discontinuation of initial T-DXd	
PD (n = 11)	11.8 (2.3–NC)
ILD, AEs other than ILD, others (n = 15)	Not reached
**Tumor response**	
**ORR, % (95% CI)**	
Overall (N = 26)	27 (11.6–47.8)
By reason for discontinuation of initial T-DXd	
PD (n = 11)	0 (0.0–28.5)
ILD (n = 4)	50 (6.8–93.2)
AEs other than ILD (n = 6)	50 (11.8–88.2)
Others (n = 5)	40 (5.3–85.3)
**BOR, n (%)**	
CR	1 (4)
PR	6 (23)
SD	5 (19)
PD	8 (31)
NE	6 (23)

Abbreviations: AE, adverse event, BOR, best overall response; CI, confidence interval; CR, complete response; ILD, interstitial lung disease; NC, not calculated; NE, not evaluable; ORR, objective response rate; OS overall survival; PD, progressive disease; PR, partial response; Q, quartile; rwPFS, real-world progression-free survival, rwTTF, real-world time to treatment failure; SD, stable disease; T-DXd, trastuzumab deruxtecan.

a Time from start of the T-DXd rechallenge.

rwPFS, rwTTF, and OS overall, by BOR to initial T-DXd, and by reason for initial T-DXd discontinuation are shown in Supplementary Fig. S1–S3 and Table 2. Patients who discontinued initial T-DXd due to PD (n = 11) had median rwPFS, rwTTF, and OS of 2.1 months (95% CI, 0.9–4.0), 2.1 months (1.2–4.0), and 11.8 months (2.3–NC), respectively. No objective responses were observed during rechallenge in this subgroup (Table 2). Conversely, among patients who discontinued initial T-DXd without PD (n = 15), median rwPFS, rwTTF, and OS were not reached, with 6-month rates of 85.6%, 79.4%, and 92.9%, respectively (Supplementary Fig. S1–S3). Objective responses were observed in 40%–50% of patients who discontinued initial T-DXd because of ILD, AEs other than ILD, or other causes (Table 2).

Discontinuation of initial T-DXd treatment due to PD was associated with numerically shorter rechallenge durations compared with discontinuation without PD (Fig. 2). T-DXd rechallenge duration exceeded that of the initial T-DXd treatment in five patients; reasons for initial T-DXd treatment discontinuation were ILD (n = 1), other AEs (n = 3), and other causes (n = 1) (Fig. 2 and Supplementary Table S3).

### 3.3 ILD incidence

No patients experienced ILD during T-DXd rechallenge, including four who had ILD during initial T-DXd treatment (grade 1, n = 3; grade 2, n = 1). In these four patients, T-DXd rechallenge durations were 4.6, 6.4, 10.2, and 11.5 months, with treatment ongoing in three patients at the data cutoff (Supplementary Table S3).

## 4 Discussion

This exploratory analysis of EN-SEMBLE showed that T-DXd rechallenge following one non-T-DXd intervening line of treatment had clinical activity in a small, selected cohort, leading to objective responses in 7/26 patients, a median rwPFS of 6.5 months, and no observed ILD during rechallenge.

Among patients who received T-DXd rechallenge, rwPFS, rwTTF, and OS were numerically longer in those who discontinued initial T-DXd treatment without PD than in those who discontinued due to PD. This finding may suggest that patients who discontinued T-DXd for reasons other than disease progression could represent a subgroup in whom rechallenge may be clinically considered. However, this observation was based on a small subgroup (n = 15) and may reflect selection bias and confounding by indication, including differences in underlying disease biology, rather than a treatment effect of rechallenge itself. Therefore, these results should be interpreted cautiously.

Patients who received T-DXd rechallenge and those who received treatments other than T-DXd differed substantially in baseline and treatment-related characteristics. Thus, direct comparison between these groups was considered inappropriate and was not performed. Additional studies are needed to better characterize patient selection and outcomes associated with T-DXd rechallenge.

No ILD was observed during T-DXd rechallenge, including among the four patients who experienced ILD during initial T-DXd treatment. However, this observation was based on a very small number of patients with prior ILD and should be interpreted as hypothesis-generating. A recent report found that ILD recurred in approximately 26% of patients with prior T-DXd-related ILD upon rechallenge [10]. Differences between that report and the present analysis may reflect small sample size, patient selection, effective clinical management, and follow-up; therefore, these findings should not be interpreted as establishing the safety of rechallenge after prior ILD.

This study has several limitations. First, the rechallenge cohort was small, clinically heterogeneous, and highly selected, representing 6% of patients who received a second post-T-DXd treatment, thus limiting the generalizability of the findings. Additionally, patients were heavily pretreated, with a median of four prior treatment lines for mBC before initial T-DXd, meaning that rechallenge occurred later in the treatment course. The observed differences by reason for initial T-DXd discontinuation may reflect treatment selection bias and differences in disease biology, including more indolent disease among patients who discontinued without PD. Furthermore, outcomes were assessed in routine clinical practice, without standardized imaging intervals or central review, and detailed information on treatment-related toxicities and T-DXd dose at rechallenge was not collected. Therefore, these findings should be interpreted as hypothesis-generating rather than a basis for defining rechallenge strategies. Nonetheless, this study addresses a clinically important question for which evidence remains limited, and contributes additional real-world experience with T-DXd rechallenge.

## 5 Conclusions

This exploratory analysis of EN-SEMBLE suggests that T-DXd rechallenge following one non-T-DXd intervening line of treatment may have clinical activity in selected, late-line patients with HER2-positive mBC. Given the small cohort size and potential selection bias, these findings, including the absence of observed ILD during rechallenge, should be interpreted cautiously.

## Meeting presentation

The results of this study have been presented as an abstract at the Japanese Society of Medical Oncology Annual Meeting in Yokohama, Japan, March 26–28, 2026.

## Data sharing

De-identified and anonymized study participants data underlying the results presented in this manuscript may be made available to researchers upon submission of a reasonable request to the corresponding author. The decision to share the de-identified/anonymized study data will be made by the corresponding author and the funder, Daiichi Sankyo Co., Ltd. Formal data sharing requests can be made up to 36 months from the article publication.

## Funding

This study was sponsored by 10.13039/501100002973Daiichi Sankyo Co., Ltd. The funding provider was involved in the study design, planning of the data analysis, data interpretation, and development of the manuscript but was not involved in the data management and statistical analysis. In March 2019, AstraZeneca entered into a global development and commercialization collaboration agreement with Daiichi Sankyo for trastuzumab deruxtecan (T-DXd; DS-8201).

## CRediT authorship contribution statement

**Kazuki Nozawa:** Conceptualization, Formal analysis, Investigation, Methodology, Project administration, Visualization, Writing – original draft, Writing – review & editing. **Hiroji Iwata:** Conceptualization, Formal analysis, Investigation, Methodology, Supervision, Visualization, Writing – original draft, Writing – review & editing. **Toru Mukohara:** Investigation, Writing – review & editing. **Tetsuhiko Taira:** Investigation, Writing – review & editing. **Akiyo Yoshimura:** Investigation, Writing – review & editing. **Shigenori E. Nagai:** Investigation, Writing – review & editing. **Jun Hashimoto:** Investigation, Writing – review & editing. **Kazuo Matsuura:** Investigation, Writing – review & editing. **Toshiro Mizuno:** Investigation, Writing – review & editing. **Yoshiaki Shinden:** Investigation, Writing – review & editing. **Mitsugu Yamamoto:** Investigation, Writing – review & editing. **Toshimi Takano:** Investigation, Writing – review & editing. **Makoto Wakahara:** Investigation, Writing – review & editing. **Hirofumi Terakawa:** Investigation, Writing – review & editing. **Takashi Yamanaka:** Investigation, Writing – review & editing. **Yasuyuki Kojima:** Investigation, Writing – review & editing. **Takahiro Nakayama:** Investigation, Writing – review & editing. **Yuji Hirakawa:** Conceptualization, Formal analysis, Investigation, Methodology, Visualization, Writing – original draft, Writing – review & editing. **Kazuhito Shiosakai:** Formal analysis, Investigation, Methodology, Writing – review & editing. **Junji Tsurutani:** Conceptualization, Formal analysis, Funding acquisition, Investigation, Methodology, Project administration, Supervision, Visualization, Writing – original draft, Writing – review & editing.

## Declaration of competing interest

All authors received support from Daiichi Sankyo Co., Ltd. for medical writing and the article processing charge for the submitted article. **Kazuki Nozawa** received lecture fees from Daiichi Sankyo Co., Ltd., related to the submitted work, and lecture fees from Eli Lilly Japan K.K., Daiichi Sankyo Co., Ltd., and Chugai Pharmaceutical Co., Ltd., outside the submitted work. **Hiroji Iwata** received consulting fees and honoraria from Daiichi Sankyo Co., Ltd., Chugai Pharmaceutical Co., Ltd., AstraZeneca K.K., Eli Lilly Japan K.K., MSD K.K., and Pfizer Japan Inc., consulting fees from Gilead Sciences, Inc., and honoraria from Taiho Pharmaceutical Co., Ltd. and Kyowa Kirin Co., Ltd., and his institution received research funding from Chugai Pharmaceutical Co., Ltd., Daiichi Sankyo Co., Ltd., and AstraZeneca K.K., outside the submitted work. **Toru Mukohara** received research funding from Sysmex Corporation, Sanofi K.K., MSD K.K., Pfizer Japan Inc., Novartis Pharma K.K., Chugai Pharmaceutical Co., Ltd., AstraZeneca K.K., Ono Pharmaceutical Co., Ltd., Daiichi Sankyo Co., Ltd., and Gilead Sciences, Inc., and lecture fees from Eisai Co., Ltd., Pfizer Japan Inc., Novartis Pharma K.K., Chugai Pharmaceutical Co., Ltd., Eli Lilly Japan K.K., AstraZeneca K.K., Kyowa Kirin Co., Ltd., Taiho Pharmaceutical Co., Ltd., and Daiichi Sankyo Co., Ltd., outside the submitted work. **Tetsuhiko Taira** received honoraria from Kyowa Kirin Co., Ltd., Chugai Pharmaceutical Co., Ltd., Eli Lilly Japan K.K., Pfizer Japan Inc., Taiho Pharmaceutical Co., Ltd., Daiichi Sankyo Co., Ltd., and MSD K.K., outside the submitted work. **Akiyo Yoshimura** received honoraria from Chugai Pharmaceutical Co., Ltd., AstraZeneca K.K., and Pfizer Japan Inc., outside the submitted work. **Shigenori E. Nagai** received research funding from Daiichi Sankyo Co., Ltd., related to the submitted work; honoraria from Eli Lilly Japan K.K., Pfizer Japan Inc., Daiichi Sankyo Co., Ltd., Chugai Pharmaceutical Co., Ltd., Eisai Co., Ltd., MSD K.K., Gilead Sciences, Inc., and Kyowa Kirin Co., Ltd., and received consulting fees as a member of advisory boards funded by Daiichi Sankyo Co., Ltd. and Chugai Pharmaceutical Co., Ltd., outside the submitted work. **Jun Hashimoto** received honoraria from Daiichi Sankyo Co., Ltd., related to the submitted work; Pfizer Japan Inc., AstraZeneca K.K., Eisai Co., Ltd., and Nippon Shinyaku Co., Ltd., outside the submitted work. **Kazuo Matsuura** received research funding from Eisai Co., Ltd., and lecture fees from Eisai Co., Ltd., Chugai Pharmaceutical Co., Ltd., Pfizer Japan Inc., Daiichi Sankyo Co., Ltd., Kyowa Kirin Co., Ltd., and AstraZeneca K.K., outside the submitted work. **Toshiro Mizuno** received research funding from Daiichi Sankyo Co., Ltd., related to the submitted work; research funding from AstraZeneca K.K., Gilead Sciences, Inc., and Novartis Pharma K.K., honoraria from AstraZeneca K.K., Pfizer Japan Inc., Ono Pharmaceutical Co., Ltd., Chugai Pharmaceutical Co., Ltd., Eli Lilly Japan K.K., MSD K.K., Eisai Co., Ltd., Novartis Pharma K.K., and Kyowa Kirin Co., Ltd., and is a member of the Data Monitoring Committee funded by Taiho Pharmaceutical Co., Ltd., outside the submitted work. **Yoshiaki Shinden** received research funding from Daiichi Sankyo Co., Ltd., related to the submitted work; and lecture fees from Daiichi Sankyo Co., Ltd., Chugai Pharmaceutical Co., Ltd., Pfizer Japan Inc., Eisai Co., Ltd., Eli Lilly Japan K.K., Takeda Pharmaceutical Co., Ltd., Novartis Pharma Co., Ltd., Nippon Kayaku Co., Ltd., AstraZeneca K.K., and Kyowa Kirin Co., Ltd., outside the submitted work. **Mitsugu Yamamoto** received lecture fees from Pfizer Japan Inc., AstraZeneca K.K., Chugai Pharmaceutical Co., Ltd., and Merck & Co., Inc., outside the submitted work. **Toshimi Takano** received honoraria from Daiichi Sankyo Co., Ltd., Chugai Pharmaceutical Co., Ltd., and Eli Lilly Japan K.K., outside the submitted work. **Makoto Wakahara** received research funding from Daiichi Sankyo Co., Ltd., related to the submitted work; and lecture fees from Daiichi Sankyo Co., Ltd., Eli Lilly Japan K.K., Eisai Co., Ltd., AstraZeneca K.K., Pfizer Japan Inc., Kyowa Kirin Co., Ltd., MSD K.K., Gilead Sciences, Inc., Chugai Pharmaceutical Co., Ltd., and Taiho Pharmaceutical Co., Ltd., outside the submitted work. **Hirofumi Terakawa** received research funding from Chugai Pharmaceutical Co., Ltd., and honoraria from Daiichi Sankyo Co., Ltd., Chugai Pharmaceutical Co., Ltd., MSD K.K., Eli Lilly Japan K.K., Taiho Pharmaceutical Co., Ltd., Kyowa Kirin Co., Ltd., and Eisai Co., Ltd., outside the submitted work. **Takashi Yamanaka** received consulting fees from Daiichi Sankyo Co., Ltd., and Eli Lilly Japan K.K.; honoraria from AstraZeneca K.K., Chugai Pharmaceutical Co., Ltd., Eli Lilly Japan K.K., Daiichi Sankyo Co., Ltd., Kyowa Kirin Co., Ltd., Pfizer Japan Inc., Taiho Pharmaceutical Co., Ltd., and Gilead Sciences, Inc., outside the submitted work. **Yasuyuki Kojima** received honoraria from Daiichi Sankyo Co., Ltd., AstraZeneca K.K., Kyowa Kirin Co., Ltd., Chugai Pharmaceutical Co., Ltd., Biomaster, Inc., Exact Sciences Corp., MSD K.K., and Taiho Pharmaceutical Co., Ltd., and lecture fees from Daiichi Sankyo Co., Ltd. and Eisai Co., Ltd., outside the submitted work. **Takahiro Nakayama** received lecture fees from Chugai Pharmaceutical Co., Ltd., Eli Lilly Japan K.K., Novartis Pharma K.K., AstraZeneca K.K., Pfizer Japan Inc., Taiho Pharmaceutical Co., Ltd., Daiichi Sankyo Co., Ltd., Eisai Co., Ltd., MSD K.K., Sandoz K.K., Celltrion Healthcare Japan K.K., Yakult Honsha Co., Ltd., and Nippon Kayaku Co., Ltd., outside the submitted work. **Yuji Hirakawa** and **Kazuhito Shiosakai** are employees of Daiichi Sankyo Co., Ltd. **Junji Tsurutani** received research funding from Daiichi Sankyo Co., Ltd., related to the submitted work; honoraria from Daiichi Sankyo Co., Ltd., AstraZeneca K.K., Eli Lilly Japan K.K., Pfizer Japan Inc., Eisai Co., Ltd., Kyowa Kirin Co., Ltd., Taiho Pharmaceutical Co., Ltd., MSD K.K., and Gilead Sciences, Inc., and consulting fees from Daiichi Sankyo Co., Ltd., AstraZeneca K.K., Seagen Inc., Pfizer Japan Inc., and Gilead Sciences, Inc., and his institution received research funding from Eli Lilly Japan K.K., Kyowa Kirin Co., Ltd., Taiho Pharmaceutical Co., Ltd., Chugai Pharmaceutical Co., Ltd., MSD K.K., and Eisai Co., Ltd., outside the submitted work.
