# Supplementary material for: Trastuzumab deruxtecan rechallenge in HER2-positive metastatic breast cancer: EN-SEMBLE
Source: Breast. 2026 Jul 22;89:104883. doi: 10.1016/j.breast.2026.104883 (PMC13486309; doi:10.1016/j.breast.2026.104883)
Supplement: Supplementary file 1 [file mmc1.docx]

**Online supplementary materials**

**Trastuzumab Deruxtecan Rechallenge in HER2-Positive Metastatic Breast Cancer: EN-SEMBLE**

Nozawa K et al.

**Supplementary Figure S1. (P2)**

rwPFS in patients who received T-DXd rechallenge

**Supplementary Figure S2. (P4)**

rwTTF in patients who received T-DXd rechallenge

**Supplementary Figure S3. (P6)**

OS in patients who received T-DXd rechallenge

**Supplementary Table S1. (P8)**

Background and clinical characteristics of individual patients who received T-DXd rechallenge (N=26)

**Supplementary Table S2. (P9)**

Demographic and clinical characteristics in patients who received treatments other than T-DXd

**Supplementary Table S3. (P11)**

Treatment outcomes and duration of treatment of individual patients who received T-DXd rechallenge (N=26)

**Supplementary Table S4. (P12)**

Second post-T-DXd treatment outcomes in patients who received treatments other than T-DXd

**Supplementary Figure S1.**

rwPFS in patients who received T-DXd rechallenge

**(a) Overall**

**(b) By best overall response to initial T-DXd treatment**

**(c) By reason for initial T-DXd discontinuation**

Abbreviations: AEs, adverse events; CI, confidence interval; CR, complete response; ILD, interstitial lung disease; mo, month; NC, not calculated; No., number; PD, progressive disease; PR, partial response; rwPFS, real-world progression-free survival; SD, stable disease; T‑DXd, trastuzumab deruxtecan.

**Supplementary Figure S2.**

rwTTF in patients who received T-DXd rechallenge

**(a) Overall**

**(b) By best overall response to initial T-DXd treatment**

**(c) By reason for initial T-DXd discontinuation**

Abbreviations: AEs, adverse events; CI, confidence interval; CR, complete response; ILD, interstitial lung disease; mo, month; NC, not calculated; No., number; PD, progressive disease; PR, partial response; rwTTF, real-world time to treatment failure; SD, stable disease; T‑DXd, trastuzumab deruxtecan.

**Supplementary Figure S3.**

OS in patients who received T-DXd rechallenge

**(a) Overall**

**(b) By best overall response to initial T-DXd treatment**

**(c) By reason for initial T-DXd discontinuation**

Abbreviations: AEs, adverse events; CI, confidence interval; CR, complete response; ILD, interstitial lung disease; mo, month; NC, not calculated; No., number; OS, overall survival; PD, progressive disease; PR, partial response; SD, stable disease; T‑DXd, trastuzumab deruxtecan.

**Supplementary Table S1.**

Background demographic and clinical characteristics of individual patients who received T-DXd rechallenge (N=26)

| **Pt no.** | **Age** | **Sex** | **HER2 status** | **HR** | **ECOG PS** | **Visceral metastasis** | **Brain metastasis** | **Number of LOT prior to initial**  **T-DXd treatment** |
| --- | --- | --- | --- | --- | --- | --- | --- | --- |
| **1** | 59 | F | IHC3+ | + | 0 | N | N | 6 |
| **2** | 66 | F | IHC3+ | - | 0 | Y | N | 6 |
| **3** | 60 | F | IHC3+ | + | 1 | Y | Y | 5 |
| **4** | 50 | F | IHC3+ | + | 0 | Y | N | 5 |
| **5** | 56 | F | IHC3+ | + | 2 | Y | Y | 3 |
| **6** | 55 | F | IHC3+ | - | 0 | Y | N | 12 |
| **7** | 75 | F | NA | + | 0 | Y | Y | 3 |
| **8** | 80 | F | IHC3+ | - | 1 | Y | N | 11 |
| **9** | 75 | F | IHC2+ / ISH+ | + | 1 | Y | N | 3 |
| **10** | 82 | F | IHC3+ | + | 0 | Y | N | 4 |
| **11** | 52 | F | IHC3+ | - | 0 | N | N | 2 |
| **12** | 49 | F | IHC3+ | + | 0 | Y | Y | 7 |
| **13** | 50 | F | IHC3+ | + | 2 | Y | Y | 2 |
| **14** | 51 | F | IHC2+ / ISH+ | + | 1 | Y | N | 1 |
| **15** | 73 | F | IHC2+ / ISH+ | + | 0 | N | N | 5 |
| **16** | 57 | F | IHC2+ / ISH+ | + | 0 | Y | N | 4 |
| **17** | 59 | F | IHC2+ / ISH+ | + | 1 | Y | N | 3 |
| **18** | 49 | F | IHC3+ | + | 2 | N | Y | 4 |
| **19** | 61 | F | IHC3+ | + | 0 | Y | Y | 5 |
| **20** | 67 | F | IHC3+ | - | NA | Y | N | 3 |
| **21** | 53 | F | IHC3+ | + | 0 | Y | Y | NA |
| **22** | 46 | F | IHC2+ / ISH+ | + | 0 | Y | N | 3 |
| **23** | 67 | F | IHC3+ | - | 0 | Y | N | 8 |
| **24** | 50 | F | IHC2+ / ISH+ | + | 1 | Y | N | 3 |
| **25** | 46 | F | IHC3+ | - | 1 | Y | Y | 5 |
| **26** | 43 | F | NA | - | NA | Y | N | 3 |

Abbreviations: ECOG PS, Eastern Cooperative Oncology Group performance status; HER2, human epidermal growth factor receptor 2; F, female; HR, hormone receptor; IHC, immunohistochemistry; ISH, in situ hybridization; LOT, lines of treatment; N, none; NA, not available; No, number; Pt, patient; T‑DXd, trastuzumab deruxtecan; Y, yes.

**Supplementary Table S2.**

Demographic and clinical characteristics in patients who received treatments other than T-DXd

| **Items** | **Treatments other than T-DXd**  **(N=383)** |
| --- | --- |
| **At the start of the first post-T-DXd treatment** |  |
| **Age** |  |
| Median (Q1, Q3), years | 59 (51, 68) |
| <65 years | 258 (67) |
| ≥65 years | 125 (33) |
| **Sex** |  |
| Female | 382 (100) |
| Male | 1 (0) |
| **Hormone receptor status** |  |
| Positive | 238 (62) |
| Negative | 137 (36) |
| Unknown/missing | 8 (2) |
| **HER2 status** |  |
| IHC3+ | 229 (60) |
| IHC2+ / ISH+ | 107 (28) |
| Other ISH+ / ISH- | 19 (5) |
| Unknown / both missing | 28 (7) |
| **ECOG PS** |  |
| 0 | 200 (52) |
| 1 | 146 (38) |
| ≥2 | 22 (6) |
| Unknown | 15 (4) |
| **Visceral metastasis** |  |
| Yes | 302 (79) |
| No | 81 (21) |
| **Brain metastasis** |  |
| Yes | 75 (20) |
| No | 303 (79) |
| Unknown | 5 (1) |
| **Prior cancer therapy for mBC before initial T-DXd treatment** |  |
| **Number of prior regimens** |  |
| Median (Q1, Q3), line | 3 (2, 5) |
| ≤2 lines | 115 (30) |
| ≥3 lines | 261 (68) |
| Unknown/missing | 7 (2) |
| **Prior regimens** |  |
| Anti-HER2 therapy | 377 (98) |
| Trastuzumab | 361 (94) |
| Pertuzumab | 350 (91) |
| T-DM1 | 348 (91) |
| HER2-TKI | 108 (28) |
| Chemotherapy | 343 (90) |
| Endocrine therapy | 153 (40) |
| **Initial T-DXd treatment** |  |
| **mBC status** |  |
| *De novo* stage IV | 165 (43) |
| Recurrent BC | 209 (55) |
| Others | 4 (1) |
| Unknown/missing | 5 (1) |
| **Duration of initial T-DXd treatment, median (Q1, Q3), months** | 7.9 (5.5, 12.8) |
| **ORR of initial T-DXd treatment** | 218 (57) |
| **BOR of initial T-DXd treatment** |  |
| CR | 10 (3) |
| PR | 208 (54) |
| SD | 122 (32) |
| PD | 37 (10) |
| NE | 6 (2) |
| **History of ILD incidence caused by initial T-DXd treatment** |  |
| Yes | 95 (25) |
| No | 287 (75) |
| Unknown | 1 (0) |
| **Reason for discontinuation of initial T-DXd** |  |
| PD | 274 (72) |
| ILD | 89 (23) |
| AEs other than ILD | 9 (2) |
| Others | 11 (3) |
| **Interval between initial T-DXd discontinuation and second post-T-DXd treatment, median (Q1, Q3), months** | 5.2 (3.5, 8.4) |

Data are shown as n (%) unless otherwise noted.

Abbreviations: AE, adverse event; BC, breast cancer; BOR, best overall response; CR, complete response; ECOG PS, Eastern Cooperative Oncology Group performance status;HER2, human epidermal growth factor receptor 2; IHC, immunohistochemistry; ILD, interstitial lung disease; ISH, in situ hybridization; mBC, metastatic breast cancer; NE, not evaluable; ORR, objective response rate; PD, progressive disease; PR, partial response; Q, quartile; SD, stable disease; T-DM1, trastuzumab emtansine; T-DXd, trastuzumab deruxtecan; TKI, tyrosine kinase inhibitor.

**Supplementary Table S3.**

Treatment outcomes and duration of treatment of individual patients who received T-DXd rechallenge (N=26)

| **Pt**  **no.** | **Initial T-DXd Tx period** | | | | **First post-T-DXd Tx period** | | | | **T-Dxd rechallenge period** | | | **Duration of treatment (mo)** | | | | | |
| --- | --- | --- | --- | --- | --- | --- | --- | --- | --- | --- | --- | --- | --- | --- | --- | --- | --- |
|  | **mBC status** | **BOR** | **RFD** | **ILD (Gr)** | **Tx** | **BOR** | **RFD** | **ILD** | **BOR** | **RFD** | **ILD** | **Initial**  **T-DXd Tx** | **No Tx**  **FU 1** | **First post-**  **T-DXd** | **No Tx**  **FU 2** | **T-DXd RC** | **Initial T-DXd discont. to RC** |
| **1** | R | PR | AE | N | Tra + ET (O) | SD | PD | N | NE | → | N | 5.5 | 5.0 | 11.7 | 0.9 | 17.5 | 17.7 |
| **2** | O | PR | AE | N | Tra + Doc + CBDCA | SD | AE | N | SD | → | N | 7.6 | 9.8 | 8.1 | 1.1 | 12.9 | 19.1 |
| **3** | D | NE | ILD | Y (1) | LET | PD | PD | N | SD | → | N | 10.6 | 0.6 | 10.6 | 0 | 11.5 | 11.4 |
| **4** | R | PR | O | N | Tra + Per | SD | PD | N | NE | → | N | 15.2 | 0.9 | 12.0 | 1.1 | 10.4 | 14.1 |
| **5** | D | PR | ILD | Y (1) | Lapa + Cape | PR | PD | N | PR | → | N | 16.4 | 1.1 | 4.2 | 0.2 | 10.2 | 5.6 |
| **6** | R | CR | O | N | Tra | SD | PD | N | PR | PD | N | 8.3 | 0 | 10.1 | 0 | 9.4 | 10.3 |
| **7** | R | NE | AE | N | Eri | CR | PD | N | PR | → | N | 1.4 | 1.7 | 14.0 | 13.3 | 8.6 | 29.2 |
| **8** | R | PR | AE | N | Lapa + Cape + Others | PD | PD | N | PR | PD | N | 13.1 | 0 | 4.1 | 0.7 | 8.5 | 4.9 |
| **9** | R | PR | AE | N | Tra | SD | PD | N | PR | → | N | 9.1 | 0 | 11.0 | 0.2 | 8.1 | 11.4 |
| **10** | R | SD | PD | N | Lapa + Cape | SD | PD | N | NE | → | N | 23.8 | 0.3 | 3.4 | 0 | 6.8 | 3.8 |
| **11** | R | PR | ILD | Y (1) | TDM-1 | PD | PD | N | PD | PD | N | 9.9 | 0.6 | 2.1 | 0.6 | 6.4 | 3.5 |
| **12** | R | PR | PD | N | Tra + Per + LET | PD | PD | N | SD | PD | N | 9.3 | 0.3 | 2.9 | 0.1 | 4.8 | 3.4 |
| **13** | R | PR | ILD | Y (2) | Tra + Per + PTX + LH-RH analog + TAM | PR | PD | N | PR | → | N | 13.6 | 3.7 | 4.1 | 0.9 | 4.6 | 8.8 |
| **14** | R | SD | O | N | T-DM1 | PD | PD | N | SD | O | N | 9.1 | 0 | 3.0 | 0.2 | 4.5 | 3.3 |
| **15** | D | CR | AE | N | Tra + Per + LET | PD | PD | N | NE | → | N | 8.5 | 12.1 | 5.3 | 0.4 | 4.1 | 17.9 |
| **16** | R | PD | PD | N | DXR + CPA | PD | PD | N | PD | PD | N | 5.2 | 0 | 1.4 | 0.4 | 4.0 | 1.9 |
| **17** | R | CR | O | N | Tra + Per + LET | PD | PD | N | PD | PD | N | 16.3 | 0.6 | 4.4 | 0.4 | 3.7 | 5.6 |
| **18** | R | SD | PD | N | Bev + PTX | PD | PD | N | PD | PD | N | 22.8 | 0.4 | 2.4 | 0.8 | 3.5 | 3.7 |
| **19** | R | CR | O | N | Tra | PR | PD | N | CR | O | N | 11.7 | 0 | 8.2 | 1.9 | 3.0 | 10.2 |
| **20** | R | PR | PD | N | Tra | NE | PD | N | NE | PD | N | 14.6 | 1.3 | 3.7 | 0.7 | 2.5 | 5.8 |
| **21** | NA | SD | PD | N | Lapa + Cape | PR | PD | N | PD | PD | N | 10.1 | 0 | 15.9 | 0.7 | 2.1 | 16.7 |
| **22** | R | PR | PD | N | Lapa + Cape | PD | PD | N | SD | PD | N | 11.0 | 0 | 2.6 | 0.5 | 1.8 | 3.3 |
| **23** | R | PR | PD | N | Tra + Per + PTX | PD | PD | N | PD | PD | N | 13.6 | 0.8 | 3.2 | 0.4 | 1.6 | 4.6 |
| **24** | R | SD | PD | N | Tra + Per | PD | PD | N | PD | PD | N | 10.5 | 0.5 | 1.8 | 0.2 | 1.6 | 2.6 |
| **25** | R | PR | PD | N | Bev + PTX | PR | PD | N | PD | PD | N | 15.0 | 0.4 | 5.4 | 0.5 | 1.2 | 6.5 |
| **26** | R | PR | PD | N | Tra + Per + Eri | PD | PD | N | NE | PD | N | 8.3 | 0.6 | 2.6 | 1.0 | 0.9 | 4.4 |

In the RFD, the arrow indicates an ongoing status.

Abbreviations: AE, adverse events other than ILD; Bev, bevacizumab; BOR, best overall response; Cape, capecitabine; CBDCA, carboplatin; CPA, cyclophosphamide; CR, complete response; D, *de novo* stage IV; Discont., discontinue; Doc, docetaxel; DXR, doxorubicin; Eri, eribulin; ET, endocrine therapy; FU, follow-up; Gr, grade; ILD, interstitial lung disease; Lapa, lapatinib; LET, letrozole; LH-RH, luteinizing hormone-releasing hormone; mBC, metastatic breast cancer; N, none; NA, not available; NE, not evaluable; No., number; O, others; PD, progressive disease; Per, pertuzumab; PR, partial response; Pt, patient; PTX, paclitaxel; R, recurrent; RC, rechallenge; RFD, reason for discontinuation; SD, stable disease; TAM, tamoxifen; T-DM1, trastuzumab emtansine; T‑DXd, trastuzumab deruxtecan; Tra, trastuzumab; Tx, treatment; Y, yes.

**Supplementary Table S4.**

Second post-T-DXd treatment outcomes in patients who received treatments other than T-DXd

| **Items** | **Treatments other than T-DXd**  **(N=383)** |
| --- | --- |
| **Follow-up period, median (Q1, Q3) months^a^** | 8.4 (4.6, 13.7) |
| **Situation at the time of data cutoff, n (%)** |  |
| Continuation | 47 (12) |
| Discontinuation | 335 (87) |
| Unknown | 1 (0) |
| **Survival outcomes, median (95% CI) months^a^** |  |
| **rwPFS** |  |
| Overall (N=383) | 3.3 (2.8–3.5) |
| By BOR of initial T-DXd |  |
| CR/PR (n=218) | 3.1 (3.0–3.7) |
| SD/PD/NE (n=165) | 3.5 (3.0–4.2) |
| By reason for discontinuation of initial T-DXd |  |
| PD (n=274) | 3.0 (2.7–3.3) |
| ILD, AEs other than ILD, others (n=109) | 4.9 (3.7–6.0) |
| **rwTTF** |  |
| Overall (N=383) | 3.2 (2.9–3.5) |
| By BOR of initial T-DXd |  |
| CR/PR (n=218) | 3.0 (2.8–3.5) |
| SD/PD/NE (n=165) | 3.5 (3.0–4.0) |
| By reason for discontinuation of initial T-DXd |  |
| PD (n=274) | 2.9 (2.6–3.2) |
| ILD, AEs other than ILD, others (n=109) | 4.9 (3.7–6.0) |
| **OS** |  |
| Overall (N=383) | 10.8 (9.8–12.7) |
| By BOR of initial T-DXd |  |
| CR/PR (n=218) | 10.6 (9.4–13.7) |
| SD/PD/NE (n=165) | 10.9 (9.3–14.9) |
| By reason for discontinuation of initial T-DXd |  |
| PD (n=274) | 9.7 (8.4–10.8) |
| ILD, AEs other than ILD, others (n=109) | 20.0 (13.0–NC) |
| **Tumor response** |  |
| **ORR, % (95% CI)** |  |
| Overall (N=383) | 9 (6.7–12.8) |
| By reason for discontinuation of initial T-DXd |  |
| PD (n=274) | 10 (6.9–14.4) |
| ILD (n=89) | 8 (3.2–15.5) |
| AEs other than ILD (n=9) | 11 (0.3–48.2) |
| Others (n=11) | 0 (0.0–28.5) |
| **BOR, n (%)** |  |
| CR | 2 (1) |
| PR | 34 (9) |
| SD | 122 (32) |
| PD | 187 (49) |
| NE | 38 (10) |

Abbreviations: AE, adverse event, BOR, best overall response; CI, confidence interval; CR, complete response; ILD, interstitial lung disease; NC, not calculated; NE, not evaluable; ORR, objective response rate; OS overall survival; PD, progressive disease; PR, partial response; Q, quartile; rwPFS, real-world progression-free survival; rwTTF, real-world time to treatment failure; SD, stable disease; T-DXd, trastuzumab deruxtecan.

^a^Time from initiation of the second post-T-DXd treatment.
